# Increased Lysis of Stem Cells but Not Their Differentiated Cells by Natural Killer Cells; De-Differentiation or Reprogramming Activates NK Cells

**DOI:** 10.1371/journal.pone.0011590

**Published:** 2010-07-16

**Authors:** Han-Ching Tseng, Aida Arasteh, Avina Paranjpe, Antonia Teruel, Wendy Yang, Armin Behel, Jackelyn A. Alva, Gina Walter, Christian Head, Tomo-o Ishikawa, Harvey R. Herschman, Nicholas Cacalano, April D. Pyle, No-Hee Park, Anahid Jewett

**Affiliations:** 1 Division of Oral Biology and Oral Medicine, School of Dentistry and Medicine, The Jane and Jerry Weintraub Center for Reconstructive Biotechnology, University of California Los Angeles, Los Angeles, California, United States of America; 2 Department of Microbiology, Immunology and Molecular Genetics (MIMG), School of Dentistry and Medicine, Broad Stem Cell Research Center (BSCRC), University of California Los Angeles, Los Angeles, California, United States of America; 3 Division of Head and Neck Surgery, Department of Surgery, School of Dentistry and Medicine, University of California Los Angeles, Los Angeles, California, United States of America; 4 School of Dentistry and Medicine, The Jonsson Comprehensive Cancer Center, University of California Los Angeles, Los Angeles, California, United States of America; 5 Department of Biological Chemistry, School of Dentistry and Medicine, University of California Los Angeles, Los Angeles, California, United States of America; 6 Department of Radiation Oncology, School of Dentistry and Medicine, University of California Los Angeles, Los Angeles, California, United States of America; New York University, United States of America

## Abstract

The aims of this study are to demonstrate the increased lysis of stem cells but not their differentiated counterparts by the NK cells and to determine whether disturbance in cell differentiation is a cause for increased sensitivity to NK cell mediated cytotoxicity. Increased cytotoxicity and augmented secretion of IFN-γ were both observed when PBMCs or NK cells were co-incubated with primary UCLA oral squamous carcinoma stem cells (UCLA-OSCSCs) when compared to differentiated UCLA oral squamous carcinoma cells (UCLA-OSCCs). In addition, human embryonic stem cells (hESCs) were also lysed greatly by the NK cells. Moreover, NK cells were found to lyse human Mesenchymal Stem Cells (hMSCs), human dental pulp stem cells (hDPSCs) and human induced pluripotent stem cells (hiPSCs) significantly more than their differentiated counterparts or parental lines from which they were derived. It was also found that inhibition of differentiation or reversion of cells to a less-differentiated phenotype by blocking NFκB or targeted knock down of COX2 in monocytes significantly augmented NK cell cytotoxicity and secretion of IFN-γ. Taken together, these results suggest that stem cells are significant targets of the NK cell cytotoxicity. However, to support differentiation of a subset of tumor or healthy untransformed primary stem cells, NK cells may be required to lyse a number of stem cells and/or those which are either defective or incapable of full differentiation in order to lose their cytotoxic function and gain the ability to secrete cytokines (split anergy). Therefore, patients with cancer may benefit from repeated allogeneic NK cell transplantation for specific elimination of cancer stem cells.

## Introduction

Immunosuppression and tumor escape from immune recognition are thought to be the two major factors responsible for the establishment and progression of cancer. A number of factors responsible for the suppression of NK cell cytotoxicity in humans have been identified previously [1], [2], [3], [4], [5], [6]. However, the significance and the precise mechanism of NK suppression induced during their interaction with either tumor cells or healthy primary cells are not well understood. It is shown that freshly isolated tumor infiltrating NK cells are not cytotoxic to autologous tumors. Moreover, NK cells obtained from the peripheral blood of patients with cancer have significantly reduced cytotoxic activity [7], [8], [9], [10]. In addition, NK cell cytotoxicity is suppressed after their interaction with stem cells [11], [12], [13]. In contrast the interaction of NK cells with the resistant tumors does not lead to suppression of NK cell cytotoxicity [14]. Many mechanisms have been proposed for the functional inactivation of tumor associated NK cells including the over-expression of Fas ligand, the loss of mRNA for granzyme B [2] and decreased CD16 and its associated zeta chain [15].

Many metastatic tumor cells exhibit constitutively elevated NFκB activity [16]. Increased NFκB activity is shown to have a causal relationship to neoplastic transformation, and uncontrolled cell growth in many cell types [16]. Human solid tumors exhibit constitutively activated NFκB [16].

We have previously shown that NK resistant primary oral keratinocyte tumors demonstrate higher nuclear NFκB activity and secrete significant levels of Granulocyte Monocyte-Colony Stimulating Factor (GM-CSF), Interleukin(IL)-1β, IL-6 and IL-8 [17]. Moreover, treatment with Non-steroidal anti-inflammatory drugs (NSAIDs) which inhibit NFκB has the ability to reverse immunosuppression induced by a tobacco-specific carcinogen, in addition to their well established ability to decrease oral dysplasia as well as induction of overt cancer in transgenic animals [18]. In agreement, we have previously demonstrated that inhibition of NFκB by Sulindac treatment of tumor cells increases functional activity of NK cells [19], [20]. Moreover, targeted inhibition of NFκB in skin epithelial cells resulted in the induction of auto-immunity and inflammation [21].

The exact mechanisms by which NFκB nuclear function in oral keratinocytes modulate and shape the function of key interacting immune effectors is yet to be determined. We have previously shown that inhibition of NFκB by the IκB super-repressor in HEp2 tumors leads to significant increase in cytotoxicity and secretion of IFN-γ by the human NK cells [19], [20]. However, neither the underlying significance nor the physiological relevance of NFκB modulation in tumors or in healthy cells responsible for the alteration of NK cell cytotoxic function have been studied previously. It is clear that the objective in cancer is to enhance the function of cytotoxic immune effectors to eliminate tumors and in auto-immunity and inflammation the aim is to inhibit immune effector function to prevent tissue damage. Therefore, dissection of the underlying mechanisms of immune activation when NFκB is modulated in the cells might help design strategies to target each disease accordingly. Indeed, targeted inhibition of NFκB function in both the intestinal epithelial cells and the myeloid cells was previously shown to result in a significant decrease in the size and the numbers of the tumor cells [22].

Here we have extended our previous results obtained by an established HEp2 oral tumor line [19] to patient derived oral tumors demonstrating that blocking NFκB in these cells increases the activation of NK cell cytotoxicity. We have also used an immortalized but non tumorigenic oral keratinocytes HOK-16B since they were previously used as a model of dysplasia in a cancer progression model [23], [24].

In this report we demonstrate that the stage of differentiation of the cells is predictive of their sensitivity to NK cell lysis. Thus, UCLA-OSCSCs, which are less differentiated oral tumors, are significantly more susceptible to NK cell mediated cytotoxicity; however, their differentiated counterparts UCLA-OSCCs are significantly more resistant. In addition, both hESCs and iPSCs as well as a number of other stem cells such as hMSCs and hDPSCs were found to be significantly more susceptible to NK cell mediated cytotoxicity. Based on these results, we propose that NK cells may play a significant role in differentiation of the cells by providing critical cytokines. However, to drive differentiation, NK cells will have to first receive signals from undifferentiated stem cells or those which have disturbed or defective capabilities to differentiate in order to lose cytotoxicity and gain in cytokine producing phenotype. These alterations in NK cell effector function will ultimately aid in driving differentiation of a sub-population of surviving healthy as well as transformed cells. In cancer patients since the majority of NK cells have lost their cytotoxic activity, they may eventually contribute rather than halt the progression of cancer by not only driving the differentiation of tumor cells but more importantly, by allowing the growth and expansion of the pool of cancer stem cells.

## Materials and Methods

### Cell Lines, Reagents, and Antibodies

RPMI 1640 supplemented with 10% FBS was used for the cultures of human and mouse NK cells and human PBMCs. UCLA-OSCCs and UCLA-OSCSCs were isolated from freshly resected tongue tumors, and were cultured in RPMI 1640 supplemented with 10% FBS. The immortalized human oral keratinocytes with type 16 human papillomavirus DNA (HOK-16B) were cultured as described previously [25] in keratinocytes growth medium (KGM) supplemented with reagents supplied in the bullet kit (Clonetics Corp., San Diego, CA). The mouse and human NK and monocyte purification kits were obtained from Stem Cell Technologies (Vancouver, Canada). Recombinant IL-2 was obtained from NIH- BRB. The anti- CD133 antibody was obtained from Miltenyi biotec (Auburn, CA). Antibodies to CD90 and CD44 were purchased from Pharmingen/BD (San Diego, CA). Antibodies to CD16 and B7H1 were purchased from ebiosciences (San Diego, CA). EGFR antibody (Erbitux) was purchased from UCLA pharmacy. The antibodies against p65 subunit of NFκB and pSTAT3 were purchased from Santa Cruz (Santa Cruz, CA).

### Human Mesenchymal stem cells (hMSCs), human Embryonic Stem cells (hESCs), human Dental Pulp Stem cells (hDPSCs), human induced pluripotent stem cells (hiPSCs)

hMSCs were obtained from Poietics, Cambrex Bio Science (Walkerville, MD) and they were cultured in Mesenchymal Stem Cell Basal Medium (MSCBM) supplemented with Mesenchymal Cell Growth Supplement (MCGS) (Cambrex Bio Science Walkerville, MD). The hMSCs were differentiated into osteoblasts using Osteogenic differentiation media which comprises of Osteogenic Differentiation BulletKit® that contains Basal Medium and one Osteogenic SingleQuot Kit® also purchased from Cambrex Bio Science (Walkerville, MD). Human Mesenchymal stem cells were cultured in Mesenchymal Stem Cell Basal Medium (MSCBM) with the growth supplements according to the manufacturer's recommendations. For the induction of osteogenesis, hMSCs were seeded at a density of (1×10^4^ cells/well) in Osteogenic media with the recommended supplements. Media was replaced every three days and the cells were used in the experiments when they were 80% confluent.

hDPSCs were isolated as described previously [26] and they were cultured in complete DMEM supplemented with 10% FBS. DPSCs were differentiated using β-glycerophosphate, ascorbic acid and dexamethasone as described previously [26].

hESC line H9 and hiPSC line hiPSC18 [27] were used at passages 45–50. hESCs and hiPSCs were grown on irradiated mouse embryonic fibroblasts (MEFs) in DMEM/F12 supplemented with 20% Knockout serum replacement (Invitrogen), 1mM glutamine, 1× nonessential amino acids (NEAA), and 4 ng/ml of bFGF as previously described [28]. 2-mercaptoethanol (1 mM Sigma) and penicillin/streptomycin (Hyclone) were added to growing cultures. For coculture assays, cells were seeded at a density of 10^5^ cells/well on Matrigel (BD Sciences) in conditioned media plus HA-1077, as previously described [28]. Neonatal human dermal fibroblasts (NHDF-iPSC parental fibroblast line from ATCC) [27] were cultured in DMEM supplemented with 10% FBS, 1mM glutamine, 1× NEAA and penicillin/streptomycin.

### Purification of human and mouse NK cells and monocytes

Written informed consents approved by UCLA Institutional Review Board (IRB) were obtained from the blood donors and all the procedures were approved by the UCLA-IRB. PBMCs and NK cells from healthy donors were isolated as described before [14]. Briefly, peripheral blood lymphocytes were obtained after Ficoll-hypaque centrifugation and purified NK cells were negatively selected by using an NK cell isolation kit (Stem Cell Technologies, Vancouver, Canada). The purity of NK cell population was found to be greater than 90% based on flow cytometric analysis of anti-CD16 antibody stained cells. The levels of contaminating CD3+ T cells remained low, at 2.4%±1%, similar to that obtained by the non-specific staining using isotype control antibody throughout the experimental procedures. The adherent subpopulation of PBMCs was detached from the tissue culture plates and monocytes were purified using isolation kit obtained from Stem Cell Technologies (Vancouver, Canada). Greater than 95% purity was achieved based on flow cytometric analysis of CD14 antibody stained monocytes. Dendritic Cells (DCs) were generated from Monocytes cultured in GM-CSF and IL-4 for 7 days before use in the experiments.

All animal work performed was based on the guidelines established and approved by UCLA-IACUC (2006-074-12). Single cell preparations of mouse splenocytes were used to negatively select for mouse NK cells using mouse NK isolation kit purchased from Stem Cell Technologies (Vancouver, Canada). The purity of mouse NK cells were greater than 90% based on staining with NK1.1 and DX5 antibodies. Murine monocytes were purified from bone marrow using monocyte isolation kit obtained from Stem Cell Technologies (Vancouver, Canada). The purity of monocytes was greater than 90% based on staining with anti-CD14 antibody.

### ELISA and Multiplex Cytokine Array kit

Single ELISAs were performed as described previously [14]. Fluorokine MAP cytokine multiplex kits were purchased from R&D Systems (Minneapolis, MN) and the procedures were conducted as suggested by the manufacturer. To analyze and obtain the cytokine concentration, a standard curve was generated by either two or three fold dilution of recombinant cytokines provided by the manufacturer. Analysis was performed using the Star Station software.

### Surface Staining

Staining was performed by labeling the cells with antibodies as described previously [29] [14], [30].

### Western Blot

Treated and untreated cells were lysed in a lysis buffer containing 50mM Tris-HCL (pH 7.4), 150mM NaCl, 1% Nonidet P-40 (v/v), 1mM sodium orthovanadate, 0.5mM EDTA, 10mM NaF, 2mM PMSF, 10µg/mL leupeptin, and 2U/mL aprotinin for 15 minutes on ice. The samples were then sonicated for 3 seconds. The cell lysates were centrifuged at 14,000 rpm for 10 minutes and the supernatants were removed and the levels of protein were quantified by the Bradford method. The cell lysates were denatured by boiling in 5× SDS sample buffer. Equal amounts of cell lysates were loaded onto 10% SDS-PAGE and transferred onto Immobilon-P membranes (Millipore, Billerica MA). The membranes were blocked with 5% non-fat milk in PBS plus 0.1% Tween-20 for 1 hour. Primary antibodies at the predetermined dilutions were added for 1 hour at room temperature. Membranes were then incubated with 1∶1000 dilution of horseradish peroxidase-conjugated secondary antibody. Blots were developed by enhanced chemiluminescence (ECL- purchased from Pierce Biotechnology, Rockford, IL).

### ^51^Cr release cytotoxicity assay

The ^51^Cr release assay was performed as described previously [20]. Briefly, different numbers of purified NK cells were incubated with ^51^Cr-labeled target cells. After a 4 hour incubation period the supernatants were harvested from each sample and counted for released radioactivity using the gamma counter. The percentage specific cytotoxicity was calculated as follows:
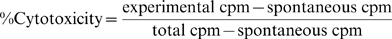



LU 30/10^6^ is calculated by using the inverse of the number of effector cells needed to lyse 30% of target cells ×100.

### Retroviral and lentiviral transduction

UCLA-OSCCs were infected with culture supernatants of NIH 3T3 packaging cells transfected with either IκB_(S32AS36A)_ super-repressor or mutant IκBα (IκBαM) or their EGFP control vectors. The retroviral vectors were generated in Dr. Nicholas Cacalano's laboratory. Forty eight hours after infection the UCLA-OSCCs or HOK-16B cells were sorted for high expressing GFP cells and were grown and used in the experiments.

NFκB-Luciferase lentiviral reporter vector was produced by co-transfection of the packaging cell line 293T [26] using Calcium Phosphate precipitation. UCLA-OSCCs and UCLA-OSCSCs were seeded at a density of 2×10^5^ cells per well in a 6-well culture plate 24hrs before transduction. The following day, cells were transduced with the NFκB-Luciferase lentiviral reporter vector. To enhance transduction efficiency, the cationic polymer Polybrene was used at a final concentration of 8µg/ml. After six hours of incubation, medium was re-freshed and transduced cells were incubated for an additional 42 hours. Cells were then harvested, lysed and luciferase activity was measured [RLU/s] using a luminometer. An internal lentiviral vector control constitutively expressing Luciferase was used to normalize values.

### Luciferase reporter assay

Transfections were also performed using NFκB Luciferase reporter vector [31] and Lipofectamine 2000 reagent (Invitrogen, CA) in Opti-MEM media (Invitrogen, CA) for 18 hours after which they were adhered to the plate overnight before different immune effectors at 1∶1 Effector to target ratios were added. The cells were then lysed with lysis buffer and the relative Luciferase activity was measured using the Luciferase assay reagent kit obtained from Promega (Madison, WI).

### Alkaline Phosphatase (ALP) staining

Human MSCs were co-cultured with and without untreated and IL-2 treated PBMCs as indicated in the result section. Cells were then washed twice with PBS and incubated with 120mM of Tris buffer (pH = 8.4) containing 0.9mM Napthol AS-M Phosphate and 1.8mM Fast Red TR (both purchased from Sigma, MO) for 30 minutes at 37°C. After 30 minute incubation, cells were washed three times with PBS and then fixed with 1ml cold ethanol (100%) for 30 minutes. The stained cultures were scanned using an Epson scanner 1250.

### Statistical analysis

An unpaired, two-tailed student t- test was performed for the statistical analysis. One way ANOVA with a Bonferroni post test was used to compare the different groups.

## Results

### Identification and characterization of patient-derived primary oral squamous cancer stem cells (UCLA-OSCSCs)

We screened a number of different primary oral squamous cell carcinomas (OSCC) derived from patients at UCLA, and selected to concentrate on two specific primary tumors based on their phenotypic characteristics and sensitivity to NK cell mediated cytotoxicity. UCLA-OSCCs were found to have higher surface expression of B7H1 and EGF-R and moderate expression of CD44 and no surface expression of CD133 whereas UCLA-OSCSCs expressed no or very low expression of B7H1, EGF-R and increased expression of CD133 and CD44^bright^ (please see Figure S1). No surface expression of MHC-Class II (data not shown) or CD90 could be seen on either tumor type. In addition, UCLA-OSCSCs secreted no or very low levels of IL-6, IL-8 and GM-CSF whereas they secreted higher levels of VEGF when compared to UCLA-OSCCs (Tables 1 and 2). Moreover, they did not express phospho-Stat3 when cultured in the presence and absence of EGF (please see Figure S1). More importantly, no or very low activity of NFκB could be detected in UCLA-OSCSCs when compared to UCLA-OSCCs (please see Figure S1). Therefore, the profiles of cytokines secreted by UCLA-OSCCs and UCLA-OSCSCs resembled those of vector alone and IκB_(S32AS36A)_ super-repressor transfected HEp2 cells respectively (Table 2) [19], [20]. Thus, UCLA-OSCSCs expressed phenotypic characteristics of oral cancer stem cells [32], [33]. Furthermore, they were smaller in size and proliferated at a much higher rate when compared to UCLA-OSCC cells (data not shown). We used these two primary oral tumors to study NK cell function.

**Table 1:** UCLA-OSCSCs similar to HEp2-IκB_(S32AS36A)_ tumor cells secreted no or lower levels of GM-CSF, IL-6 and IL-8.

	GM-CSF	IL-6	IL-8
	pg/ml (MFI*)	pg/ml (MFI)	pg/ml (MFI)
**HEp2-vec**	0±0 (30)	20.6±1 (565)	685±20 (1390)
**HEp2-IκB_(S32AS36A)_**	0±0 (29)	1.5±0 (67)	17±0 (453)
**UCLA-OSCCs**	19.8±2 (79)	58.4±3 (1554)	906.3±50 (7583)
**UCLA-OSCSCs**	0±0 (32)	0±0 (11)	245.2±12 (3247)

HEp2-vec, HEp2-IκB_(S32AS36A)_, UCLA-OSCCs, and UCLA-OSCSCs were cultured at 1×10^5^ cells/ml and the constitutive levels of secreted GM-CSF, IL-6, and IL-8 were determined using multiplex ELISA array kit. The concentrations of secreted cytokines were determined using the standard curve for each cytokine.

*Mean fluorescence intensity (MFI). One of three representative experiments is shown.

**Table 2:** Increased ratios of IL-6 to IFN-γ secretion in NK resistant UCLA-OSCCs when compared to NK sensitive UCLA-OSCSCs.

Tumor cells	+/− Immune cells	GM-CSF	IL-8	VEGF	IL-6	IFN-γ	
		pg/ml	pg/ml	pg/ml	pg/ml	pg/ml	Ratio IL-6/IFN-γ
**UCLA-OSCCs**	**− NK**	20	438.4	620.4	126	0.8	**-**
**UCLA-OSCCs**	**+ NK (−IL-2)**	148.8	723.2	784	215	1	**215**
**UCLA-OSCCs**	**+ NK (+IL-2)**	565.8	282.2	145.5	179	820	**0.22**
**UCLA-OSCSCs**	**− NK**	0.1	23.3	1745	13	1	**-**
**UCLA-OSCSCs**	**+ NK (−IL-2)**	25	66.7	1256	65	1	**12.5**
**UCLA-OSCSCs**	**+ NK (+IL-2)**	1068.9	12.5	158	12	1730.6	**0.007**
**No tumors**	**+ NK (−IL-2)**	0.8	0	0.4	11	0.6	**18**
**No tumors**	**+ NK (+IL-2)**	403.2	3.14	8.6	13	290	**0.44**

NK cells (1×10^6^/ml) were left untreated or treated with IL-2 (1000 units/ml) for 12–24 hours before NK cells (1×10^5^/ml) were added to primary oral tumors at an effector to target ratio of 1∶1. Tumor cells were each cultured alone or in combination with NK cells as indicated in the table and the supernatants were removed from the cultures after an overnight incubation. The levels of cytokine secretion were determined using antibody coated multiplex microbead immunoassay. For simplification of the table standard deviations are not included and they ranged from 0% to a maximum of 5% of the amount obtained for each cytokine. One of three representative experiments is shown.

### Increased NK cell cytotoxicity against UCLA-OSCSCs but not those of UCLA-OSCCs

**Figure 1:**
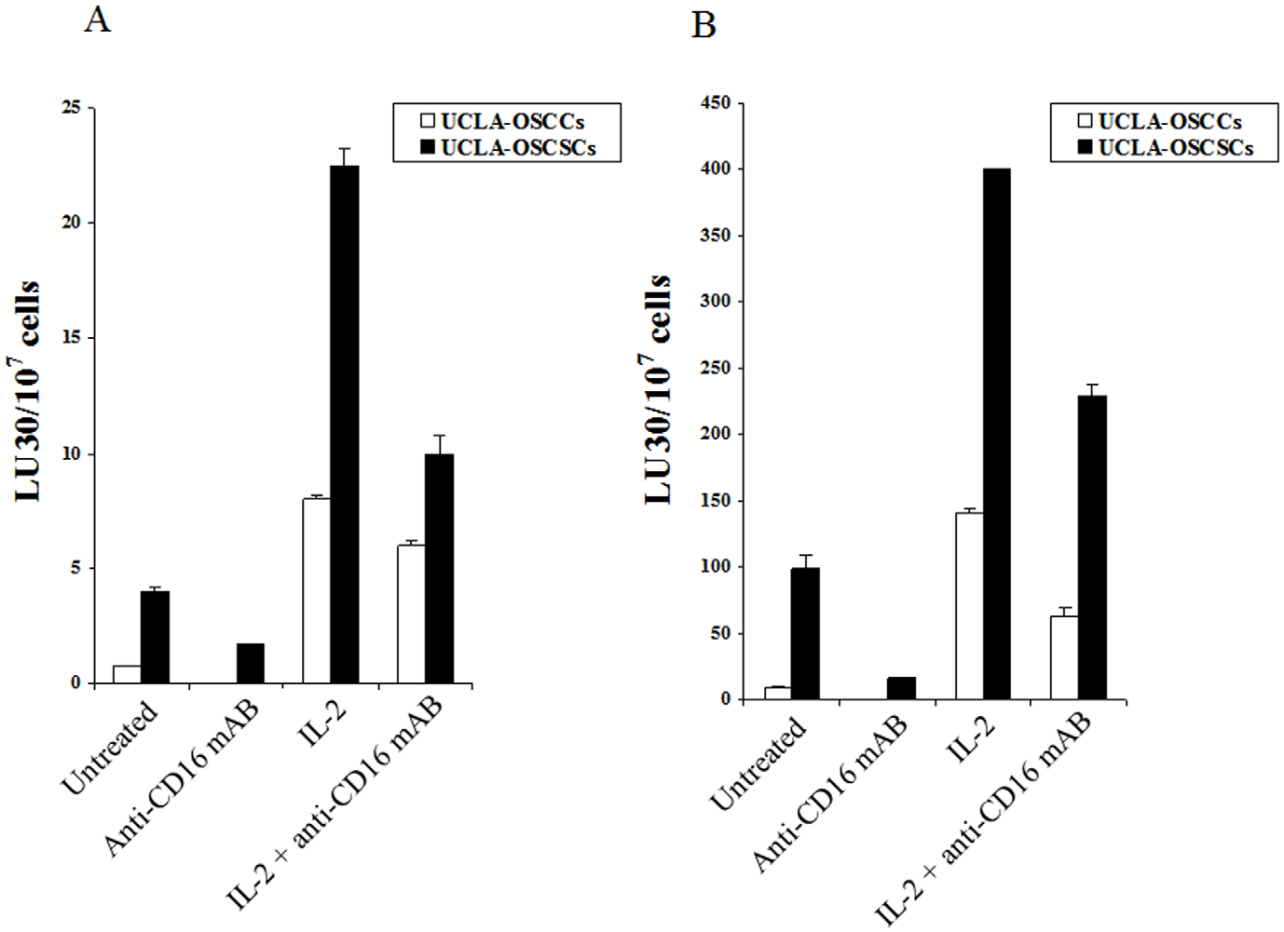
Increased NK cell cytotoxicity against UCLA-OSCSCs. PBMCs and NK cells were left untreated or treated with IL-2 (1000 units/ml) or anti-CD16 mAb (3µg/ml) or a combination of IL-2 (1000 units/ml) and anti-CD16 mAb (3µg/ml) for 12–24 hours before they were added to ^51^Cr labeled primary oral tumors. PBMC (**A**) and NK cell (**B**) cytotoxicities were determined using a standard ^51^Cr release assay and the lytic units 30/10^6^ cells were calculated using inverse number of effectors required to lyse 30% of the tumor cells ×100. Differences between untreated, anti-CD16 mAb treated or IL-2 and/or anti-CD16 mAb treated NK cell cytotoxicity between UCLA-OSCCs and UCLA-OSCSCs were significant at a p value of <0.05. One of four representative experiments is shown in this figure.

We have previously shown that blocking NFκB in HEp2 tumor cells decreased IL-6 and IL-8 secretion substantially and resulted in an increased sensitivity of HEp2 tumor cells to NK cell mediated cytotoxicity [19], [20]. Therefore, using the levels of cytotoxicity, IFN-γ and IL-6 secretion, we could demonstrate a direct correlation between decreased IL-6 and increased IFN-γ secretion in the co-cultures of NK cells with NFκB knock down HEp2 cells and increased susceptibility to IL-2 activated NK cell killing. Induction of NK cell anergy by anti-CD16 antibody, even though abrogated the ability of IL-2 treated NK cells to lyse HEp2 cells, the same treatment resulted in a significant induction of IFN-γ secretion in the co-cultures of NK cells with HEp2 cell transfectants [34], [35]. To extend our findings to patient derived oral tumors, UCLA-OSCC and UCLA-OSCSCs were tested for their sensitivity or resistance to NK cell mediated cytotoxicity. The cytotoxic activities of IL-2 treated PBMCs (Fig. 1A) and NK cells (Fig. 1B) were significantly higher against UCLA-OSCSCs when compared to UCLA-OSCCs. Untreated PBMCs or NK cells lysed UCLA-OSCSCs significantly more than UCLA-OSCCs (Fig. 1). However, the levels of lysis by untreated NK cells were considerably lower than that obtained by IL-2 treated PBMCs or NK cells (Fig. 1A and 1B). Treatment of PBMCs or NK cells with anti-CD16 mAb decreased cytotoxicity significantly against both tumor types, however, the levels of lysis by the NK cells remained higher against UCLA-OSCSCs in all the NK samples tested (Fig. 1). IL-2 treated NK cells co-cultured with UCLA-OSCSCs exhibited higher expression of CD69 activation antigen when compared to those co-cultured with UCLA-OSCCs (data not shown).

### Increased induction of IFN- γ was paralleled with a decreased secretion of IL-6 in co-cultures of NK cells with UCLA-OSCSCs

Untreated and IL-2 treated NK cells were co-cultured with UCLA-OSCCs and UCLA-OSCSCs and the induction of a number of key cytokines, including those which were correlated with NK resistant tumor phenotype, were determined in the supernatants recovered from the co-cultures of the NK cells with oral tumors after an overnight incubation. In the supernatants of untreated NK cells co-cultured with UCLA-OSCCs, synergistic induction of GM-CSF, IL-6 and IL-8 could be observed since much lower levels of these cytokines were induced either in the presence of NK cells alone or tumor cells alone (Table 2). The levels of above-mentioned cytokines were considerably lower in the co-cultures of untreated NK cells with UCLA-OSCSCs (Table 2). Even though VEGF secretion was significantly higher in UCLA-OSCSCs, the levels exceeded that of the baseline levels produced by the tumor cells alone when untreated NK cells were co-cultured with UCLA-OSCCs and not that of UCLA-OSCSCs (Table 2). Increased GM-CSF secretion in the presence of UCLA-OSCCs as compared to UCLA-OSCSCs was more evident in untreated NK cells (Table 2).

NK cell sensitivity of tumors correlated with an increased IFN-γ secretion in the presence of lower IL-6 and IL-8 secretion in IL-2 activated NK cells co-cultured with UCLA-OSCSCs (Table 2). Indeed, when ratios of IL-6 to IFN-γ was considered a direct correlation between increased sensitivity to NK cell mediated killing and decreased ratios of IL-6 to IFN-γ could be seen (Table 2). Finally, both cell lines exhibited lower amounts of VEGF secretion in the presence of IL-2 treated NK cells, indicating the ability of IL-2 treated NK cells to exert significant inhibitory effect on VEGF secretion. However, the residual levels of VEGF remained higher in the co-cultures of IL-2 treated NK cells with UCLA-OSCCs than UCLA-OSCSCs when compared to the baseline secretion by the tumors alone (Table 2). Thus, several important cytokine profiles were identified for NK sensitive and resistant oral tumors after their co-culture with NK cells.

### Blocking NFκB in UCLA-OSCCs and HOK-16B oral epithelial cells lowered IL-6 to IFN-γ ratios and increased their sensitivity to NK cell mediated cytotoxicity

**Figure 2:**
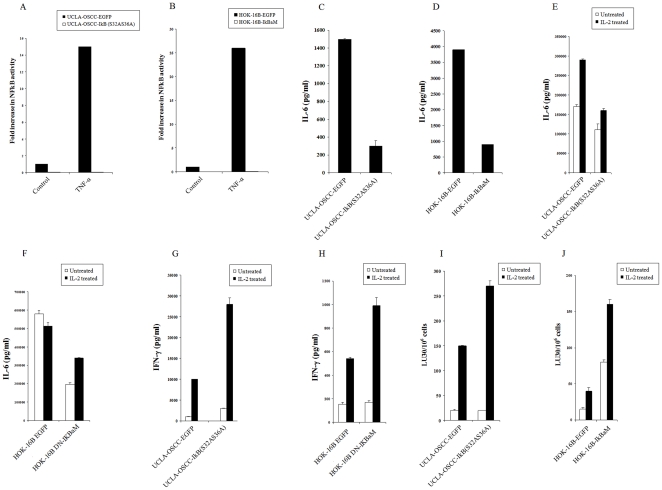
Increased cytotoxicity, decreased secretion of IL-6 and increased secretion of IFN-γ in co-cultures of NK cells with NFκB knock down UCLA-OSCCs and HOK-16B cells. IκB _(S32AS36A)_ transduced UCLA-OSCCs (**A**) and IκBαM transduced HOK-16B cells (**B**) and their EGFP transduced controls were transfected with 8 µg of NFκB Luciferase reporter vector and treated with and without TNF-α (20ng/ml) for 18 hours. The relative Luciferase activity was then determined in the lysates according to the manufacturer's recommendation and fold induction in luciferase activity was determined relative to untreated cells. IκB _(S32AS36A)_ transduced UCLA-OSCCs (**C**) and IκBαM transduced HOK-16B cells (**D**) and their EGFP transduced controls were cultured at 2×10^5^ cells/ml, and after an overnight incubation the supernatants were collected and the levels of secreted IL-6 were determined using ELISA specific for IL-6. IκB _(S32AS36A)_ transduced UCLA-OSCCs and IκBαM transduced HOK-16B cells and their EGFP transduced controls were co-cultured with untreated or IL-2 (1000 u/ml) treated NK cells at 1∶1 effector to target ratio. After an overnight incubation the supernatants from the co-cultures of UCLA-OSCCs and HOK-16B cells with NK cells were collected and the levels of secreted IL-6 (**E and F**), and IFN-γ (**G and H**) were determined by specific ELISAs for each cytokine. NK cells were left untreated or treated with IL-2 for 12–24 hours before they were added to IκB _(S32AS36A)_ transduced UCLA-OSCCs and IκBαM transduced HOK-16B cells and their EGFP transduced controls. Differences between EGFP transduced and those with either IκB _(S32AS36A)_ transduced UCLA-OSCCs or IκBαM transduced HOK-16B cells were significant for IL-2 treated NK cells at a p value of <0.05. IκB _(S32AS36A)_ transduced UCLA-OSCCs and IκBαM transduced HOK-16B cells and their EGFP transduced controls were ^51^Cr labeled before they were co-cultured with untreated or IL-2 (1000 u/ml) treated NK cells. After 4 hours of incubation at 37C cytotoxicity of NK cells were assessed using a standard ^51^Cr release assay (**I and J**). lytic unit 30/10^6^ cells were determined using inverse number of effectors required to lyse 30% of the tumor cells ×100. Differences between IκB _(S32AS36A)_ transduced UCLA-OSCCs or IκBαM transduced HOK-16B cells and those with EGFP transduced were significant in IL-2 treated PBMCs at a p value of <0.05. One of three representative experiments is shown in this figure.

As indicated previously UCLA-OSCCs and HOK-16B oral keratinocytes represent an oral cancer progression model since HOK-16B are immortalized but non tumorigenic thus could represent a model of dysplastic keratinocytes [17], [23], [24], [35]. HOK-16B and UCLA-OSCCs were transduced with EGFP alone or IκBαM or IκB_(S32AS36A)_ super-repressor retroviral constructs and sorted for high GFP expressing cells using flow cytometry (data not shown). The inhibition of NFκB by the IκBαM or IκB_(S32AS36A)_ super-repressor retroviral vector in UCLA-OSCCs and HOK-16B cells was confirmed by measuring NFκB activity using luciferase reporter assay (Figs. 2A and 2B). IκBαM or IκB_(S32AS36A)_ super-repressor transduced UCLA-OSCCs (Fig. 2C) and HOK-16B (Fig. 2D) cells secreted substantially lower levels of IL-6 when compared to EGFP transduced UCLA-OSCCs and HOK-16B cells. Thus, transduction of UCLA-OSCCs and HOK-16B cells with IκBαM or IκB_(S32AS36A)_ super-repressor constructs exhibited the same functional profiles as those seen in transfected HEp2 oral tumor cells with IκB_(S32AS36A)_ super-repressor construct [19], [20]. Similar to HEp 2 cell transfectants, UCLA-OSCCs and HOK-16B cells transduced with IκBαM or IκB_(S32AS36A)_ super-repressor constructs did not exhibit elevated levels of cell death when assessed by flow cytometric analysis of Annexin V and PI stained cells (data not shown). In addition, there was a significant decrease in the surface expression of ICAM-1 in TNF-α and IFN-γ treated IκB_(S32AS36A)_ super-repressor transduced UCLA-OSCCs (83% decrease) and HOK-16B cells (78% decrease) when compared to EGFP alone transduced cells. These results also indicated that IL-6 secretion in oral tumor cells is regulated by the function of NFκB.

Untreated or IL-2 treated NK cells were added to EGFP or IκB_(S32AS36A)_ super-repressor transduced UCLA-OSCCs and IκBαM transduced HOK-16B oral keratinocytes and the levels of IL-6 and IFN-γ secretion were determined in the co-cultures with the NK cells after an overnight incubation. IL-2 activated NK cells secreted lower levels of IL-6 when co-cultured with IκB_(S32AS36A)_ super-repressor transduced UCLA-OSCCs (Fig. 2E) and IκBαM HOK-16B (Fig. 2F) cells as compared to EGFP transduced oral keratinocytes. In contrast, higher induction of IFN-γ secretion could be observed in supernatants recovered from the co-cultures of NK cells with IκB_(S32AS36A)_ super-repressor transduced UCLA-OSCCs (Fig. 2G) and IκBαM transduced HOK-16B (Fig. 2H) oral keratinocytes as compared to EGFP transduced cells. Similar NK cell response patterns were obtained when NFκB was inhibited in HEp2 cells [19], [20]. Finally, IL-2 treated NK cells lysed NFκB knock down OSCCs (Fig. 2I) and HOK-16B (Fig. 2J) cells significantly more than EGFP transfected cells.

### Significant lysis of human Embryonic Stem Cells (hESCs), human Induced Plueripotent Stem Cells (hiPSCs), human Dental Pulp Stem Cells (hDPSCs), and human Mesenchymal Stem Cells (hMSCs) by untreated or IL-2 treated NK cells

**Figure 3:**
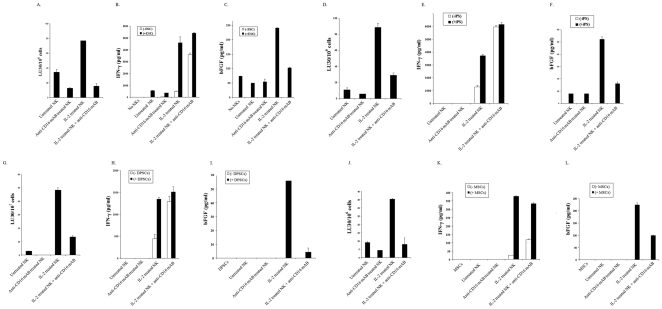
Lysis of hESCs, hiPSCs, hDPSCs and hMSCs by untreated and IL-2 treated NK cells is inhibited by anti-CD16 antibody treatment, however, the same treatment induced significant secretion of IFN-γ by the NK cells. NK cells (1×10^6^/ml) were left untreated or treated with IL-2 (1000 units/ml), or anti-CD16 mAb (3µg/ml) or a combination of IL-2 (1000 units/ml) and anti-CD16 mAb (3µg/ml) for 12-24 hours before they were added to ^51^Cr labeled hESCs, hiPSCs, hDPSCs and hMSCs. NK cell cytotoxicities were determined using a standard 4 hour ^51^Cr release assay, and the lytic units 30/10^6^ cells were determined using inverse number of NK cells required to lyse 30% of hESCs (**A**), hiPSCs (**D**), hDPSCs (**G**) and hMSCs (**J**) ×100. NK cells were treated as described above and each NK sample at (1×10^5^/ml) were either cultured in the absence or presence of hESCs, hiPSCs, hDPSCs and hMSCs at an NK to stem cell ratio of 1∶1. After an overnight culture, supernatants were removed from the co-cultures and the levels of IFN-γ (**B**,**E**,**H**,**K**), and bFGF (**C**,**F**,**I**,**L**) secretion were determined using specific ELISAs. One of a minimum three representative experiments for each stem cell population is shown in this figure.

Highly purified human NK cells were cultured with and without IL-2 for 12-24 hours before they were added to ^51^Cr labeled hESCs (Fig. 3A), hiPSCs (Fig. 3D), hDPSCs (Fig. 3G) and hMSCs (Fig. 3J). Addition of untreated NK cells had lower cytotoxicity against different populations of stem cells whereas activation with IL-2 increased cytotoxicity against all stem cell populations significantly (p<0.05) (Fig. 3). Therefore, human stem cells are greatly lysed by the NK cells.

### Lysis of hESCs, hiPSCs, hDPSCs, and hMSCs by untreated and IL-2 treated NK cells is inhibited by anti-CD16 antibody treatment, however, the same treatment induced significant secretion of IFN-γ by the NK cells in the presence and absence of stem cells

As shown in a number of previous studies and in this report anti-CD16 mAb treatment induced anergy in a great majority of NK cells as well as death in a subset of NK cells, thereby inhibiting NK cell cytotoxicity against different populations of stem cells (p<0.05) (Figs. 3). Addition of the combination of IL-2 and anti-CD16 treatment also induced anergy and NK cell death (data not shown) and inhibited significantly the NK cell cytotoxicity against stem cells when compared to IL-2 activated NK cells (p<0.05) (Fig. 3). Untreated or anti-CD16 mAb treated NK cells did not secrete IFN-γ when co-cultured with any of the stem cell populations; however, both IL-2 treated and IL-2 in combination with anti-CD16 mAb treated NK cells in the presence and absence of stem cells secreted significant levels of IFN-γ (p<0.05) (Figs. 3B, 3E, 3H, and 3K). Indeed, stem cells triggered significant secretion of IFN-γ from IL-2 treated NK cells when compared to IL-2 treated NK cells in the absence of stem cells. In addition, there was a synergistic induction of IFN-γ secretion in IL-2 and anti-CD16 mAb treated NK cells in the absence of stem cells, and the levels either plateaued or exceeded those in the absence of stem cells when IL-2 and anti-CD16mAb treated NK cells were cultured with stem cells (Fig. 3). There was a direct correlation between secretion of bFGF by stem cells and cytotoxicity by IL-2 and IL-2+anti-CD16 mAb treated NK cells (Figs. 3C, 3F, 3I, and 3L).

### Lysis of hMSCs by untreated and IL-2 treated NK cells is inhibited by monocytes, however, the addition of monocytes induced significant secretion of IFN-γ by the NK cells in the presence and absence of stem cells

**Figure 4:**
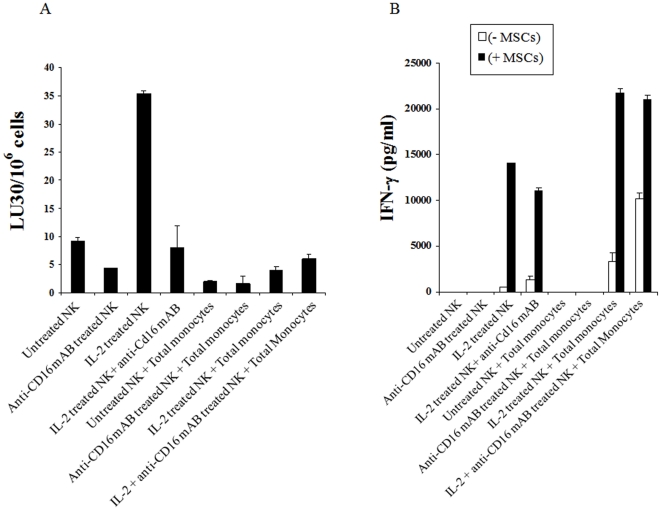
Monocytes decrease the lysis of hMSCs by the NK cells, but significantly augment the secretion of IFN-γ in the co-cultures of NK, monocyte and hMSCs. HMSCs (1×10^6^ cells/plate) were cultured with the irradiated monocytes (10 Gy) (monocyte: MSC ratio of 1∶1) for 24–48 hours before they were removed from the plates, washed and labeled with ^51^Cr and used as targets in the cytotoxicity assays against NK cells. The NK samples were either left untreated or treated with anti-CD16 mAb (3µg/ml), IL-2 (1000 u/ml), or a combination of IL-2 (1000 u/ml) and anti-CD16 mAb (3µg/ml) for 24–48 hours before they were added to ^51^Cr labeled hMSCs at different effector to target (E∶T) ratios. Supernatants were removed after 4 hours of incubation and the released radioactivity counted by a β counter. % cytotoxicities were determined at different E∶T ratio, and LU_30_/10^6^ cells were calculated using the inverse of the number of effectors needed to lyse 30% of the hMSCs ×100.. One of three representative experiments is shown in this figure (**A**). hMSCs (1×10^5^ cells/well) were co-cultured with and without irradiated Monocytes at 1∶1 hMSCs to monocytes for 24–48 hours before untreated or IL-2 (1000 u/ml) pre-treated or anti-CD16 mAb (3µg/ml) pre-treated, or a combination of IL-2 (1000 u/ml) and anti-CD16 mAb (3µg/ml) pre-treated NK cells at 1∶1∶1 NK∶monocyte∶hMSC ratios were added. NK cells were pre-treated as indicated for 24–48 hours before they were added to the co-cultures of monocytes and hMSCs. NK samples were also cultured in the absence of monocytes and hMSCs. After 24–48 hours of the addition of NK cells the supernatants were removed from the cultures and the levels of IFN-γ secretion were determined using a specific ELISA. One of five representative experiments is shown in this figure (**B**).

Monocytes were purified from PBMCs and irradiated (10 Gy) immediately before they were co-cultured with hMSCs for 24–48 hours before they were labeled with ^51^Cr and used in the cytotoxicity assays against NK cells. NK cells were left untreated or pre-treated with anti-CD16 antibody and/or IL-2 for 24–48 hours before they were used in the cytotoxicity assays against hMSCs. The addition of monocytes to hMSCs significantly protected the hMSCs (Fig. 4A) from NK cell mediated cytotoxicity (p<0.05). Significant inhibition of NK cell cytotoxicity by monocytes could be observed against untreated or IL-2 treated NK cells (p<0.05) (Fig 4A). Monocytes also increased the levels of alkaline phosphatase (ALP) staining in MSCs and prevented the decrease in ALP expression induced by IL-2 activated NK cells (data not shown). Untreated or anti-CD16 antibody treated irradiated monocytes did not mediate cytotoxicity against hMSCs, (data not shown). Overall, these experiments indicated that monocytes protect hMSCs against NK cell mediated lysis.

As expected IL-2 treated NK cells secreted moderate amounts of IFN-γ which was synergistically increased when co-cultured in the presence of hMSCs (p<0.05) (Fig. 4B). The addition of anti-CD16 mAb in combination with IL-2 to NK cells in the absence of hMSCs increased secretion of IFN-γ when compared to IL-2 alone treated NK cells in the absence of hMSCs. IFN-γ secreted levels remained similar between IL-2 alone and IL-2 and anti-CD16 mAb treated NK cells cultured with hMSCs (Fig. 4B). Monocytes added to IL-2 or IL-2 and anti-CD16 antibody treated NK cells in the absence of hMSCs or those in the presence of hMSCs, synergistically increased the levels of secreted IFN-γ (p<0.05) (Fig. 4B). However, the highest increase in IFN-γ release was seen when monocytes were added to IL-2 or IL-2 and anti-CD16 mAb treated NK cells with hMSCs (Fig. 4B). These results indicated that monocytes increased IFN-γ in co-cultures with hMSCs, and further synergized with IL-2 or IL-2 and anti-CD16 mAb treated NK samples to increase the release of IFN-γ in the co-cultures of NK cells with hMSCs. Similar results were obtained when NK cells were co-cultured with monocytes and hDPSCs [36].

### HMSCs are significantly more sensitive to lysis by IL-2 treated NK cells than their differentiated counterparts and they trigger significant release of IFN-γ by IL-2 activated NK cells

**Figure 5:**
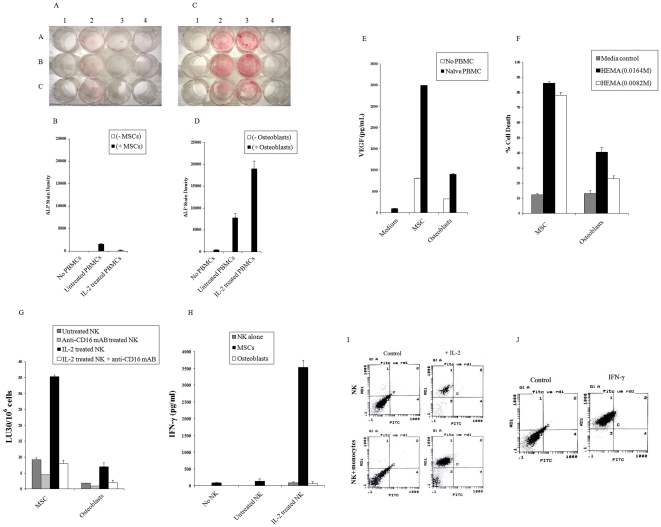
hMSCs are significantly more sensitive to lysis by IL-2 treated NK cells than their differentiated counterparts and they trigger significant release of IFN-γ by IL-2 activated NK cells. hMSCs were seeded at 3 to 4×10^5^ cells per well in Stem cell medium in the presence and absence of untreated PBMCs or IL-2 (1000u/ml) treated PBMCs (PBMC to Stem cell ratio 10∶1). After 2 days of co-cultures, Alkaline Phosphatase staining was performed. A1 to C1 (triplicates of hMSCs in the absence of PBMCs), A2 to C2 (hMSC in the presence of untreated PBMCs), A3 to C3 (MSC in the presence of IL-2 treated PBMCs), A4 (untreated PBMCs alone), B4 (IL-2 treated PBMCs alone) (**A**). The ALP stain densities for each well were determined using photoshop software (**B**). hMSCs were cultured in differentiation medium for 1 week and differentiated Osteoblasts were then seeded at 3 to 4×10^5^ cells per well in the presence and absence of untreated PBMCs and IL-2 (1000u/ml) treated PBMCs (PBMC to Stem cell ratio 10∶1). After 2 days of co-cultures Alkaline Phosphatase staining was performed. A1 to C1 (triplicates of Ostoblastic cells in the absence of PBMCs), A2 to C2 (Ostoblastic cells in the presence of untreated PBMCs), A3 to C3 (Ostoblastic cells in the presence of IL-2 treated PBMCs), A4 (untreated PBMCs alone), B4 (IL-2 treated PBMCs alone) (**C**). The ALP stain densities for each well were determined using photoshop software (**D**). hMSCs and Osteoblasts were cultured with and without untreated PBMCs as described above and after two days of incubation the supernatants were removed and subjected to specific ELISA for VEGF (**E**). NK cells (1×10^6^/ml) were left untreated or treated with IL-2 (1000 units/ml), or anti-CD16 mAb (3µg/ml) or a combination of IL-2 (1000 units/ml) and anti-CD16 mAb (3µg/ml) for 12–24 hours before they were added to ^51^Cr labeled hMSCs or osteoblasts, and NK cell cytotoxicities were determined using a standard 4 hour ^51^Cr release assay, and the lytic units 30/10^6^ cells were determined using inverse number of NK cells required to lyse 30% of the hMSCs or osteoblasts ×100 (**F**). Undifferentiated hMSCs and those differentiated to osteoblasts at (1×10^5^/ml) were cultured in the absence and presence of untreated NK cells or IL-2 treated NK cells at 1∶1 ratio, and after two days of incubation the supernatants were removed and subjected to specific ELISA for IFN-γ (**G**). HMSCs at (1×10^5^/ml) were either cultured with untreated NK cells or IL-2 treated NK cells alone (1∶1; hMSC∶NK) or with untreated NK and IL-2 treated NK cells with monocytes at (1∶1∶1; hMSC∶NK∶monocytes). After an overnight incubation, the cells were washed and B7H1 surface expression was determined on hMSC gated populations. Isotype control antibodies were used as controls (**H**). HMSCs were left untreated or treated with IFN-γ (500u/ml). After an overnight incubation, hMSCs were washed and the B7H1 surface expression was determined on hMSC (**I**).

To determine whether differentiation decreases sensitivity of stem cells to NK cell mediated cytotoxicity we first chose to concentrate on hMSCs. To assess whether differentiation of hMSCs similar to oral tumors decreases sensitivity of these cells to NK cell mediated cytotoxicity we determined NK cell cytotoxicity against hMSCs and their differentiated osteoblasts using un-fractionated PBMCs as well as NK cells. hMSCs were cultured in the absence and presence of untreated and IL-2 treated PBMCs at 10∶1 PBMC to hMSC ratio and the levels of ALP staining were determined after 2 days of incubation. The addition of untreated PBMCs to hMSCs triggered some differentiation of hMSCs as assessed by ALP staining (Figs. 5A and 5B). No significant staining with ALP can be seen by either the PBMCs or MSCs alone (Figs. 5A and 5B). Treatment of PBMCs with IL-2 and their subsequent co-culture with hMSCs lysed the cells and prevented induction of ALP, therefore, no or very low detection of ALP could be observed (Figs. 5A and 5B). The co-culture of differentiated osteoblasts with PBMCs was performed as described above with hMSCs. As shown in Figs. 5C and 5D both the untreated and IL-2 treated PBMCs triggered significant increase in ALP staining in osteoblasts. IL-2 treated PBMCs triggered much higher levels of ALP staining when compared to untreated PBMCs (Figs. 5C and 5D). The levels of ALP staining in osteoblasts were substantially lower in the absence of PBMCs and no significant ALP staining could be seen in untreated or IL-2 treated PBMCs in the absence of osteoblasts (Figs. 5C and 5D). These results suggested that stem cells were sensitive to lysis by IL-2 treated PBMCs whereas their differentiated counterparts were more resistant, and unlike stem cells they were able to resist death and further upregulate ALP expression when cultured with IL-2 treated PBMCs. In addition, when the levels of VEGF secretion were determined higher induction of VEGF secretion by hMSCs could be observed when compared to osteoblasts (Fig. 5E).

Undifferentiated hMSCs were significantly more sensitive to lysis by IL-2 treated NK cells when compared to their differentiated counterparts (Fig. 5F), and triggered significant secretion of IFN-γ in co-cultures with IL-2 treated NK cells (Fig. 5G). Moreover, when hMSCs were cultured with IL-2 treated NK cells alone or IL-2 treated NK cells with monocytes significant induction of B7H1 surface expression could be observed in surviving hMSCs (Fig. 5H). Since monocytes increase survival of hMSCs, accordingly, more surviving hMSCs was observed in co-cultures with NK cells and monocytes than with NK cells alone [36]. Monocytes alone or untreated NK cells in the presence or absence of monocytes were not able to elevate B7H1 expression on the surface of hMSCs (data not shown). The intensity of NK cell induced B7H1 expression on hMSCs were similar to that induced by the treatment of hMSCs with IFN-γ (Fig. 5I). Thus, these results suggested that sensitivity of hMSCs to NK cell mediated cytotoxicity correlated with the degree of differentiation of these cells. Moreover, it indicated that NK cells may contribute to differentiation and resistance of hMSCs by increased induction of key resistance factors such as B7H1.

### Differentiated hDPSCs are more resistant to NK cell mediated cytotoxicity

**Figure 6:**
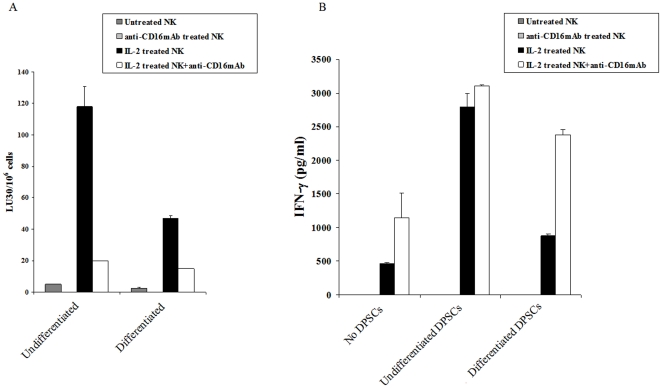
Undifferentiated DPSCs are significantly more sensitive to lysis by IL-2 treated NK cells and trigger increased secretion of IFN-γ from the NK cells than their differentiated counterparts. NK cells (1×10^6^/ml) were left untreated or treated with IL-2 (1000 units/ml), or anti-CD16 mAb (3µg/ml) or a combination of IL-2 (1000 units/ml) and anti-CD16 mAb (3µg/ml) for 12–24 hours before they were added to ^51^Cr labeled undifferentiated and differentiated DPSCs, and NK cell cytotoxicities were determined using a standard 4 hour ^51^Cr release assay. Lytic units 30/10^6^ cells were determined using inverse number of NK cells required to lyse 30% of the hDPSCs ×100. Passage 8 differentiated and undifferentiated hDPSCs were used (**A**). Undifferentiated hDPSCs and those differentiated to odontoblasts at (1×10^5^/ml) were cultured with and without untreated NK cells or IL-2 (1000 units/ml), or anti-CD16 mAb (3µg/ml) or a combination of IL-2 (1000 units/ml) and anti-CD16 mAb (3µg/ml) treated NK cells at 1∶1 ratio and after two days of incubation the supernatants were removed and subjected to specific ELISA for IFN-γ (**B**). One of three representative experiments is shown in this figure.

HDPSCs were differentiated to odontoblasts by the addition of β-glycerophosphate, ascorbic acid and dexamethasone as reported previously [26], and NK cell cytotoxicities were determined against both the differentiated and undifferentiated hDPSCs. As shown in Fig. 6 significantly less NK cell cytotoxicity as well as IFN-γ secretion could be obtained against differentiated hDPSCs by untreated, IL-2 treated and IL-2 plus anti-CD16 mAb treated NK cells when compared to undifferentiated hDPSCs. Therefore, depending on the stage of the differentiation of hDPSCs different levels of NK cell cytotoxicity can be observed against hDPSCs.

### Decreased sensitivity of dendritic cells to NK cell mediated lysis

**Figure 7:**
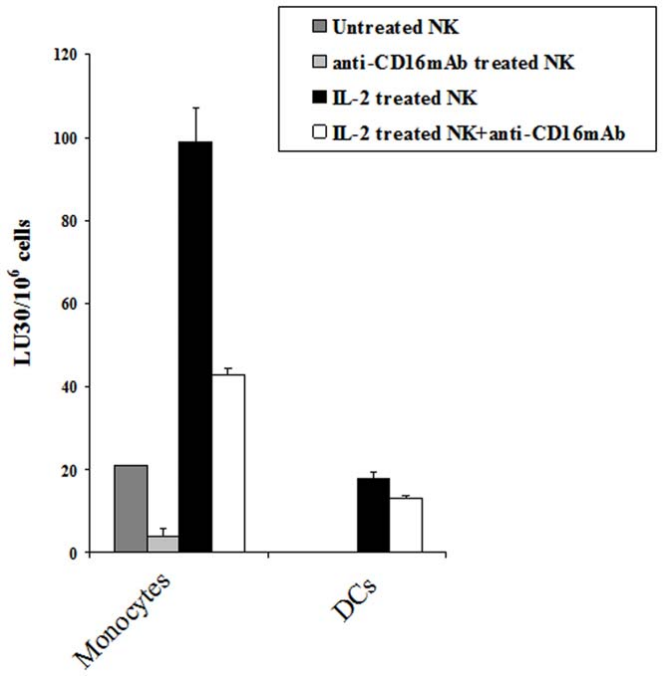
Monocytes are significantly more sensitive to NK cell mediated cytotoxicity than DCs. NK cells (1×10^6^/ml) were left untreated or treated with IL-2 (1000 units/ml), or anti-CD16 mAb (3µg/ml) or a combination of IL-2 (1000 units/ml) and anti-CD16 mAb (3µg/ml) for 12-24 hours before they were added to ^51^Cr labeled autologous monocytes or ^51^Cr labeled autologous DCs, and NK cell cytotoxicities were determined using a standard 4 hour ^51^Cr release assay and the lytic units 30/10^6^ cells were determined using inverse number of NK cells required to lyse 30% of the monocytes or DCs ×100. One of four representative experiments is shown in this figure.

To demonstrate that resistance of NK cell mediated cytotoxicity by increased differentiation of stem cells is not restricted to only certain types of cells, we used monocytes and their differentiated counterpart dendritic cells to determine sensitivity to NK cell mediated lysis. As shown in Fig. 7 monocytes were significantly more sensitive to NK cell mediated cytotoxicity than DCs.

### HiPSCs are more susceptible to NK cell mediated cytotoxicity than their parental line

**Figure 8:**
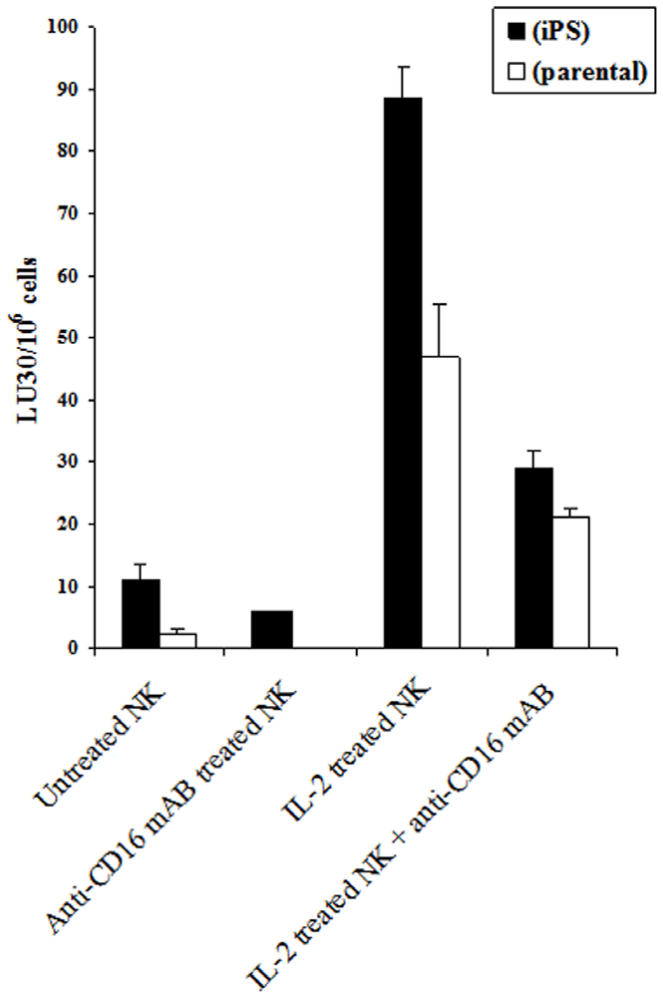
hiPSCs are more susceptible to NK cell mediated cytotoxicity than their parental line. NK cells (1×10^6^/ml) were left untreated or treated with IL-2 (1000 units/ml), or anti-CD16 mAb (3µg/ml) or a combination of IL-2 (1000 units/ml) and anti-CD16 mAb (3µg/ml) for 12–24 hours before they were added to ^51^Cr labeled hiPSCs or ^51^Cr labeled parental cells from which the hiPSCs were derived, and NK cell cytotoxicities were determined using a standard 4 hour ^51^Cr release assay and the lytic units 30/10^6^ cells were determined using inverse number of NK cells required to lyse 30% of the hiPSCs or parental cells ×100. One of two representative experiments is shown in this figure.

Since more differentiated cells were less sensitive to NK cell mediated lysis, we aimed at characterizing the sensitivity of iPSCs as well as their parental line to NK cell mediated lysis. As shown in Fig. 8 untreated or IL-2 treated NK cells lysed iPSCs significantly more than their parental line. Treatment of NK cells with anti-CD16 mAb or a combination of IL-2 and anti-CD16 mAb decreased cytotoxicity mediated by the NK cells (Fig. 8). Therefore taken together the results shown thus far suggest that any attempt in re-programming or de-differentiating the cells may result in increased sensitivity of the cells to NK cell mediated lysis. We, therefore, performed additional experiments using mice which had targeted knock down of COX2 gene in myeloid subsets to determine whether blocking COX2 which is shown to be elevated in many tumors and is important in differentiation of the cells can elevate sensitivity to NK cell mediated lysis.

### Targeted inhibition of COX2 in bone marrow derived monocytes from *LysMCre+/−* mice increased cytotoxicity and secretion of IFN-γ by IL-2 treated NK cells

**Figure 9:**
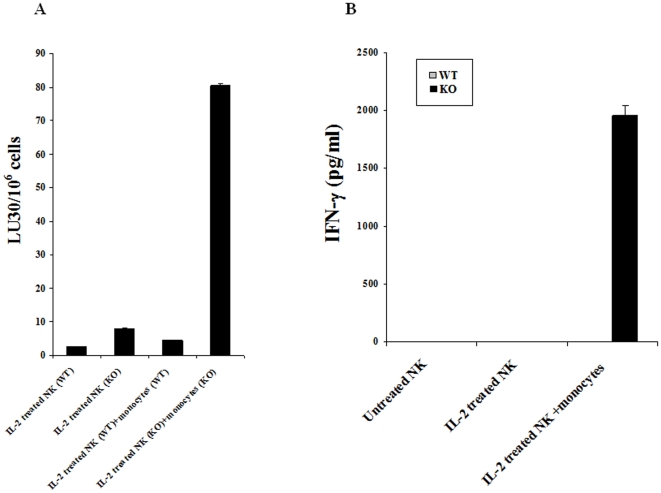
Targeted inhibition of COX2 in bone marrow monocytes increased NK cell cytotoxicity and secretion of IFN-γ by IL-2 treated NK cells. Purified NK cells and monocytes were obtained from spleens and bone marrows of 3 pooled control mice and those with targeted knock down of COX2 gene in myeloid cells respectively (n = 3). Purified NK cells and monocytes from control mice and those with targeted knock down of COX2 gene in myeloid cells were then cultured with IL-2 (1000u/ml) at 1∶1 NK: monocyte ratios for 6 days before they were added to ^51^Cr labeled YAC cells, and NK cell cytotoxicities were determined in 4 hour ^51^Cr release assay. The lytic units 30/10^6^ cells were determined using inverse number of NK cells required to lyse 30% of the YAC cells ×100 (**A**). NK cells were cultured as described in Fig. 9A and after 6 days of incubation the supernatants were removed and IFN-γ secretion were measured in the supernatants using a specific ELISA (**B**). One of five representative experiments is shown in this figure.

Purified NK cells obtained from spleens of control mice and those with targeted knock down of COX2 gene in myeloid cells [37] were cultured with and without bone marrow derived purified monocytes for 6 days before they were added to ^51^Cr YAC cells and cytotoxicity were determined in 4 hours ^51^Cr release assay. As shown in Fig. 9A NK cells purified from *Cox-2flox/flox LysMCre/+* mice and cultured with autologous COX2−/− monocytes lysed YAC cells significantly more, whereas NK cells from control mice (*Cox-2flox/flox LysM+/+*) cultured with autologous COX2+/+ monocytes had very little cytotoxicity. Similarly, NK cells purified from *Cox-2flox/flox LysMCre/+* mice and cultured with autologous COX2−/− monocytes secreted higher levels of IFN-γ when compared to NK cells from control mice (*Cox-2flox/flox LysM+/+*) cultured with autologous COX2+/+ monocytes (Fig. 9B).

## Discussion

We have characterized the interaction of two primary oral tumors and a transformed but non-tumorigenic oral keratinocyte line with NK cells and identified several important profiles which could distinguish between differentiated NK resistant oral tumors from undifferentiated NK sensitive tumor stem cells. The results also indicated that the levels of NK cell cytotoxicity may vary depending on the expression and function of NFκB in tumors. Thus, increased NFκB appears to be an important factor of differentiation, survival and function of primary oral tumors during their interaction with NK cells.

Increased NK cell cytotoxicity and augmented secretion of IFN-γ were observed when NK cells were co-incubated with UCLA-OSCSCs which released significantly lower levels of GM-CSF, IL-6 and IL-8 (Tables 1 and 2) and demonstrated decreased expression of phospho-Stat3, B7H1 and EGFR, and much lower constitutive NFκB activity when compared to differentiated UCLA-OSCCs (please see Figure S1). More importantly, UCLA-OSCSCs expressed CD133 and CD44^bright^ oral stem cell markers (please see Figure S1). Addition of untreated fresh NK cells to UCLA-OSCCs, which were unable to lyse the tumor cells, synergistically contributed to the elevation of the above mentioned cytokines in the co-cultures of NK cells with UCLA-OSCCs. In contrast, untreated NK cells, which lysed UCLA-OSCSCs, were either unable to increase or moderately increased the secretion of resistant factors in the co-cultures of NK cells with UCLA-OSCSCs. Untreated NK cells increased the secretion of VEGF in NK-UCLA-OSCC co-cultures whereas a decrease in VEGF secretion was observed in NK- UCLA-OSCSCs co-cultures when compared to those secreted by the tumors alone. Although the majority of secreted cytokines were elevated in UCLA-OSCCs when compared to UCLA-OSCSCs, the levels of VEGF secretion were higher in UCLA-OSCSCs when compared to UCLA-OSCCs. This observation is in agreement with the previously published results demonstrating decreased secretion of VEGF during the progression of head and neck tumors [38].

Increase in IFN-γ secretion was correlated with a decrease in secretion of IL-6 in co-cultures of NK cells with UCLA-OSCSCs when compared to UCLA-OSCCs. Furthermore, IL-2 activated NK cells suppressed significantly the secretion of VEGF from tumor cells. Therefore, from these results a specific profile for NK resistant oral tumors emerged which demonstrated increased GM-CSF, IL-6 and IL-8 secretion in the context of decreased IFN-γ secretion during their interaction with the NK cells. In contrast, co-cultures of cancer stem cells with NK cells demonstrated increased IFN-γ in the context of lower GM-CSF, IL-6 and IL-8 secretion.

\Many aggressive and metastatic tumor cells exhibit constitutively elevated NFκB activity [16]. Similar to HEp2 cells [19] blocking NFκB in UCLA-OSCCs and HOK-16B cells increased IFN-γ secretion and augmented the cytotoxic function of IL-2 activated NK cells against these cells (Fig. 2). Inhibition of NFκB in UCLA-OSCCs and HOK-16B was confirmed by several observations. First, the synergistic induction of ICAM-1 by TNF-α and IFN-γ treatment, which was previously shown to be due to increased function of NFκB [20], was greatly abrogated when UCLA-OSCCs and HOK-16B cells were transduced with I|B super-repressor. Second, significant decrease in IL-6 secretion could be observed in both cells and in the co-cultures of immune effectors with UCLA-OSCCs and HOK-16B cells transduced with IκB super-repressor. Lastly, decreased binding of NFκB was observed using luciferase reporter assay in NFκB knock down cells. Therefore, some of the profiles of NFκB knock down cells resembled those of undifferentiated UCLA-OSCSCs based on the parameters tested.

It appears that NFκB in primary oral keratinocytes may serve as the master molecular switch between IL-6 and IFN-γ secretion in the co-cultures of NK cells with tumors. IL-6 is secreted constitutively by oral squamous cell carcinomas [39], [40] and it is found to be elevated in oral cancer patients [39], [41]. IL-6 is known to interfere with IFN-γ signaling by the induction of Th2 differentiation via activation of NFAT which subsequently inhibits Th1 polarization [41], [42]. IL-6 is also known to induce Stat3 activation. Since blocking Stat3 function in tumor cells is also known to activate adaptive immunity [42], [43] it may be that IL-6 induced Stat3 is in part responsible for no/low activation of NK cells in the co-cultures of NK cells and either HEp2 cells or UCLA-1 or HOK-16B tumors. These possibilities are currently under investigation in our laboratory.

Since UCLA-OSCSCs were significantly more susceptible to NK cell mediated cytotoxicity we hypothesized that healthy, untransformed primary stem cells may in general be more susceptible to NK cell mediated cytotoxicity. We show in this paper that NK cells lyse hMSCs, hDPSCs, hESCs and iPSCs significantly. Taken together these results indicated that undifferentiated cells are targets of NK cell cytotoxicity. However, once NK cells lyse a proportion of sensitive targets they lose their cytotoxic function and gain the ability to secrete cytokines (split anergy) required to support differentiation of the cells not lysed by the NK cells. Indeed, similar to NK cells cultured with undifferentiated sensitive tumor stem cells or primary untransformed stem cells, the treatment of NK cells with IL-2 and anti-CD16 mAb resulted in the loss of cytotoxicity, gain in IFN-γ secretion and down modulation of CD16 surface receptors [14], [29]. Loss of cytotoxicity and gain in cytokine secretion was also seen when NK cells were cultured with hMSCs and hDPSCs in the presence of monocytes (Fig. 4) and [36].

In vivo physiological relevance of above-mentioned observations could be seen in a subpopulation of NK cells in peripheral blood, uterine and liver NK cells which express low or no CD16 receptors, and have decreased capacity to mediate cytotoxicity and is capable of secreting significant amounts of cytokines [44], [45]. Indeed, 70% of NK cells become CD16 dim or negative immediately after an allogeneic or autologous bone marrow transplantation [44]. Since NK cells lose their cytotoxic function and gain in cytokine secretion phenotype and down modulate CD16 receptors after their interaction with tumor cells or healthy primary stem cells [14], [29], it is tempting to speculate that in vivo identified CD16- NK cells and in vitro tumor induced CD16- NK cells may have similar developmental pathways since they have similar if not identical functional properties.

**Figure 10:**
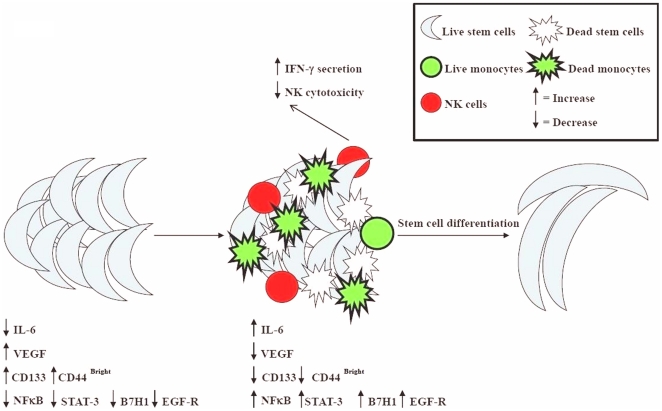
Schematic representation of hypothetical model of oral cancer stem cell or untransformed primary stem cell differentiation by NK cells and monocytes. Interaction of cancer stem cells or primary untransformed stem cells with monocytes and NK cells results in the loss of NK cell cytotoxicity due partly to the induction of resistance of cancer stem cells or primary stem cells by monocytes and indirectly by monocytes serving as targets of NK cells [36], thereby, protecting the stem cells from lysis by the NK cells. Loss of NK cell cytotoxicity by monocytes and gain in secretion of IFN-γ results in a significant induction of transcription factors, cytokines and growth factors in stem cells and differentiation of stem cells.

Since undifferentiated cells are targets of NK cells, it is logical that NFκB knock down cells are found to be more susceptible to NK cell mediated cytotoxicity since this process may revert the cells to a relatively less differentiated state and be the cause of activation of NK cells. Indeed, any disturbance in the process of differentiation should in theory result in an increase in the sensitivity of the targets to NK cell mediated cytotoxicity since this process is important for modifying the phenotype of NK cells to cytokine secreting cells in order to support differentiation of the remaining viable competent cells. In this regard knocking down COX2 in monocytes is likely the cause of reversion or de-differentiation of the monocytes and the activation of NK cell cytotoxicity. Thus, the stage of differentiation of the cells is predictive of the susceptibility of the cells to NK cell mediated cytotoxicity. In this regard we have also found higher sensitivity of hiPSCs to NK cell mediated lysis when compared to the parental line from which they were derived. In addition, hMSCs not only become resistant to NK cell mediated cytotoxicity after differentiation, but also their level of differentiation increases when they are cultured with the NK cells. As shown here co-culture of NK, monocytes and stem cells are found to result in decreased lysis of stem cells, increased secretion of IFN-γ by the NK cells and elevation of B7H1 surface expression on the stem cells (Figs. 4 and 5). Thus, stem cells which survive should exhibit differentiation markers such as increase in NFκB and STAT3 and augmented secretion of GM-CSF, IL-6 and IL-8 after interaction with NK cells and monocytes (Fig. 10).

Based on the results presented in this paper it is tempting to speculate that NK cells may have two significant functions; one that relates to the removal of unwanted stem cells that are either defective or disturbed or in general more in numbers than required for the regeneration of damaged tissue. Therefore, the first task of NK cells is to select stem cells that are competent and are able to fully differentiate to required tissues. The second important task of NK cells is to support the differentiation of the selected cells after altering the NK phenotype to cytokine secreting cells. This process will not only remove cells that are either infected or transformed, but also it will ensure the regeneration of damaged or defective tissues. Therefore, in processes in which suboptimal differentiation and regeneration of the tissues are obtained, a chronic inflammatory process may be established causing continual tissue damage and recruitment of stem cells and NK cells. Indeed, a generalized inflammatory condition in patients with Nemo mutations has been described previously, and mice with the knockdown of NFκB develop skin pathologies similar to that of inflammatory skin disease [21], [46].

The inability of cancer patient NK cells to kill cancer stem cells due to flooding of NK cells by proliferating cancer stem cells and conversion of NK cells to cytokine secreting cells may likely be a mechanism by which cancer stem cells remain viable and proliferate. Therefore, there should be two distinct strategies by the NK cells to eliminate tumors, one which targets stem cells and the other which targets differentiated cancer cells. In theory this should be achieved in oral cancer patients by the use of EGFR antibody since this antibody should target the differentiated oral tumors whereas stem cells should be eliminated by the activated NK cells. However, since the great majority of patient NK cells have modified their phenotype to support differentiation of the cells, they may not be effective in eliminating cancer stem cells. Therefore, cancer stem cells may accumulate and eventually result in the demise of the patient. These patients may therefore, benefit from the repeated allogeneic NK cell transplantation for elimination of cancer stem cells.

## Supporting Information

Figure S1 Phenotypic characteristics of UCLA-OSCCs and UCLA-OSCSCs. UCLA-OSCCs or UCLA-OSCSCs were detached, washed and stained with the antibodies specific to surface receptors indicated in the figure and analyzed by flow cytometry. Isotype control antibodies were used as controls. The numbers on the right-hand corner are the mean channel fluorescence intensity. (A). UCLA-OSCCs or UCLA-OSCSCs were left untreated or treated with EGF (10 ng/ml), and the cell extracts were prepared after an overnight incubation, and run on polyacrylamide gel, after which the bands were transferred and blotted with the antibody specifc for phospho-Stat3 (B). UCLA-OSCCs or UCLA-OSCSCs at a density of 2×105 cells per well were transduced with the NFκB-Luciferase lentiviral reporter vector for 48 hours before they were lysed and luciferase activity measured [RLU/s] using the luminometer. An internal lentiviral vector expressing constitutive Luciferase was used for normalization (C). One of three representative experiments is shown in this figure.
(2.63 MB TIF)
