# Supplementary figures and images for: Increased Lysis of Stem Cells but Not Their Differentiated Cells by Natural Killer Cells; De-Differentiation or Reprogramming Activates NK Cells
Source: PLoS One. 2010 Jul 16;5(7):e11590. doi: 10.1371/journal.pone.0011590 (PMC2905395; doi:10.1371/journal.pone.0011590)

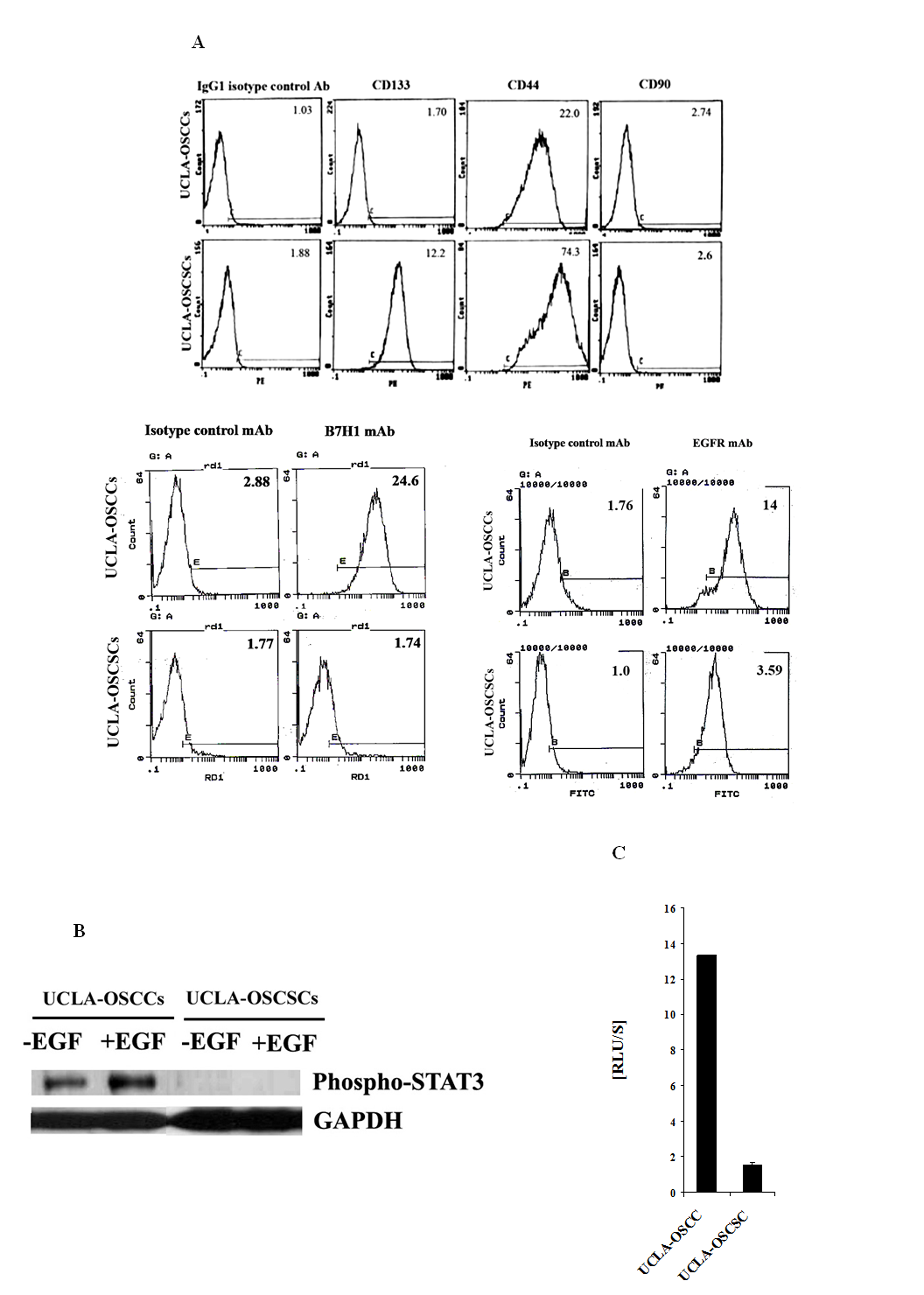

Supplement: Figure S1 — Phenotypic characteristics of UCLA-OSCCs and UCLA-OSCSCs. UCLA-OSCCs or UCLA-OSCSCs were detached, washed and stained with the antibodies specific to surface receptors indicated in the figure and analyzed by flow cytometry. Isotype control antibodies were used as controls. The numbers on the right-hand corner are the mean channel fluorescence intensity. (A). UCLA-OSCCs or UCLA-OSCSCs were left untreated or treated with EGF (10 ng/ml), and the cell extracts were prepared after an overnight incubation, and run on polyacrylamide gel, after which the bands were transferred and blotted with the antibody specifc for phospho-Stat3 (B). UCLA-OSCCs or UCLA-OSCSCs at a density of 2×10^5^ cells per well were transduced with the NFκB-Luciferase lentiviral reporter vector for 48 hours before they were lysed and luciferase activity measured [RLU/s] using the luminometer. An internal lentiviral vector expressing constitutive Luciferase was used for normalization (C). One of three representative experiments is shown in this figure. (2.63 MB TIF) [file pone.0011590.s001.tif]
